# *Microcystis* Rising: Why Phosphorus Reduction Isn’t Enough to Stop CyanoHABs

**DOI:** 10.1289/ehp.125-A34

**Published:** 2017-02-01

**Authors:** Sharon Levy

**Affiliations:** Sharon Levy, based in Humboldt County, CA, has covered ecology, evolution, and environmental science since 1993. She is at work on the book *The Marsh Builders: Wetlands in the Fight for Clean Water*.

At Stone Laboratory, Ohio State University’s island research station in western Lake Erie, a series of otherworldly life forms parades across a video monitor. Stars, double-sided combs, a web of green hexagons, triangles merged with translucent rays that form a living mandala: These are the lake’s phytoplankton seen under high magnification. *Microcystis*, the organism that dominates the harmful algal blooms (HABs) that plague Lake Erie each summer, shows up onscreen as clumps of spherical cells.

**Figure:**
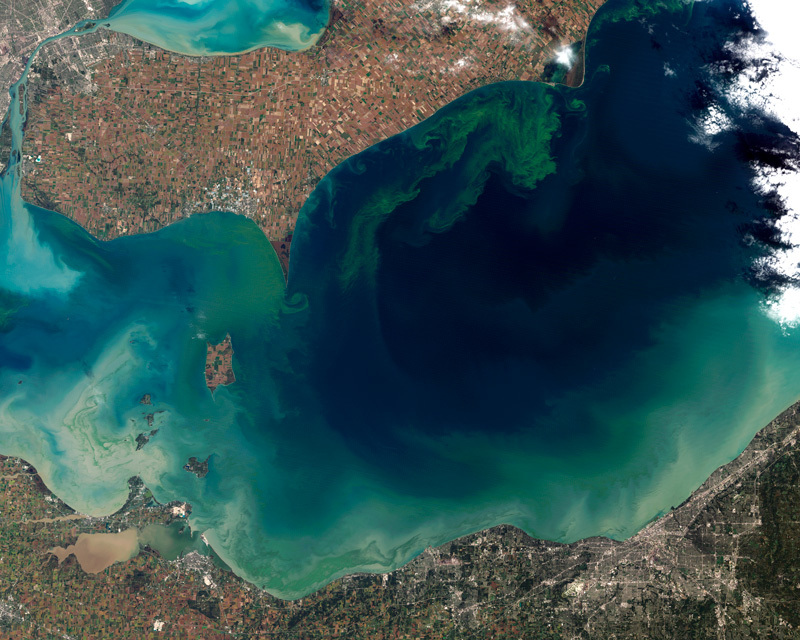
Toxic *Microcystis* blooms have become a regular summertime event on Lake Erie; this 2011 bloom was particularly bad. *Microcystis* is well adapted to the high nutrient loads that affect Erie and other lakes and estuaries. Researchers are studying the role of nitrogen in controlling the growth and toxin production of this hardy cyanobacterium. Image courtesy of NASA

For the people who live on Lake Erie’s shores, *Microcystis* made its presence most acutely known in August 2014, when a bright green patch of bloom-impacted water spread from Maumee Bay along the Ohio shoreline of western Lake Erie. Fueled by an overload of nutrients running off farm fields in the Maumee watershed, the bloom flowed over the water intake for the city of Toledo. Elevated levels of microcystins, liver toxins produced by *Microcystis*, forced city officials to distribute a “do not drink” advisory for nearly 500,000 residents. Stores ran out of bottled water, and residents fled Toledo.

Even more widespread and longer-lasting *Microcystis* blooms occurred in Lake Erie in 2011 and 2015 following intense spring rains that washed phosphorus and nitrogen into the lake, although those blooms did not affect drinking water. Any warming in temperature or increase in heavy spring rains in the Great Lakes region would be a recipe for more frequent and larger algal blooms, but of all HAB-forming species *Microcystis* would likely benefit the most.^1^ ^,^ ^2^

**Figure:**
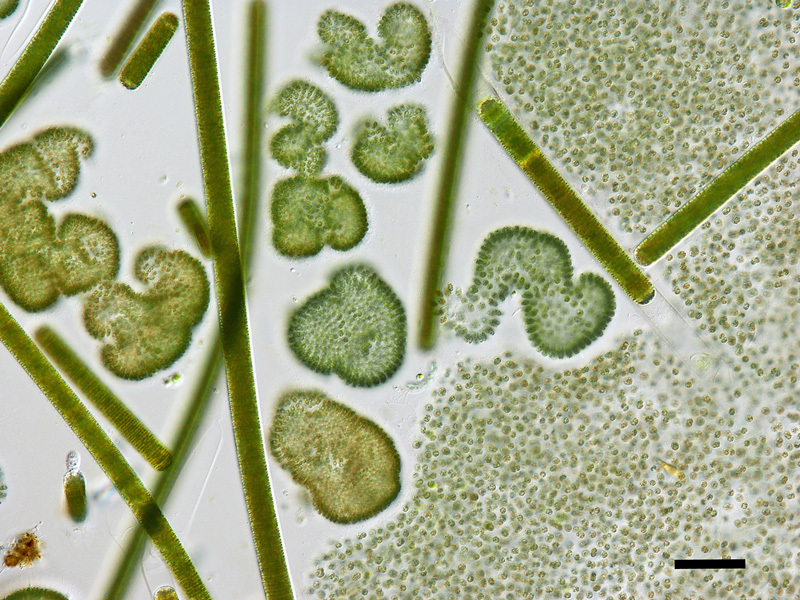
In this micrograph, *Microcystis aeruginosa* cells appear on the right as tiny green dots. Other bloom-forming cyanobacteria include *Woronichinia naegeliana* (the darker colonies shown in the center and left) and *Limnoraphis birgei* (long filaments composed of a series of cells within sheaths). Scale bar = 50 μm. © John D. Wehr/Fordham University

Traditional approaches to managing HABs have focused on controlling phosphorus levels in water. However, new insights into *Microcystis* ecology challenge long-standing ideas about how best to control these particular blooms. Human-generated phosphorus loads do fuel HABs in Lake Erie and elsewhere, but researchers now understand that an excess of another nutrient, nitrogen, shifts the balance in favor of *Microcystis* rather than other HAB-forming cyanobacteria, diatoms, or green algae.

“*Microcystis* relies on nitrogen from the watershed,” says Hans Paerl, a microbial ecologist at the University of North Carolina at Chapel Hill. “Many lakes that have *Microcystis* blooms are receiving increasing loads of nitrogen from synthetic fertilizers, urban runoff, and atmospheric pollution. Nitrogen is the new part of the story.”

## *Microcystis* on Top

Although cyanobacteria are often referred to as “blue-green algae,” they are not, in fact, algae. Similarly, although blooms of *Microcystis* and other cyanobacteria species may be lumped in with other HABs, they are more properly known as cyanobacterial HABs, or cyanoHABs.

Cyanobacteria are actually far more ancient than algae, having appeared more than 2.5–3 billion years ago.^3^ They were the first organisms to evolve photosynthesis, and their proliferation and release of great volumes of oxygen are believed to have profoundly changed the chemical makeup of Earth’s atmosphere.^3^

“Cyanobacteria have been through extreme geochemical and climate changes,” notes Paerl. “Their playbook is very deep. They’ve adapted to many of the extremes we’re seeing in the Anthropocene—excessive nutrient loads, global warming, record droughts, and extremely heavy rainfall events.”

*Microcystis* has the ability to outcompete other kinds of phytoplankton. It appears immune to predation by the planktonic crustaceans, such as *Daphnia*, that usually control populations of green algae and diatoms.^2^ These “grazers” avoid *Microcystis* cells, perhaps because they are less able to devour the clumps of cells. In experiments, daphnids seem unaffected by microcystins, which are deadly to vertebrates, but grazers may be put off by other chemicals produced by *Microcystis*, including protease inhibitors that can halt digestion.^1^

*Microcystis* is also rejected by zebra mussels, which rapidly spread throughout Lake Erie after they were inadvertently introduced in the 1980s via ballast water. Zebra mussels are filter feeders that devour algae, and by 1996 they had drastically reduced most phytoplankton populations to 20% of their pre-invasion biovolume (the abundance of cells in an amount of water).^4^ Zebra mussels spit *Microcystis* cells back into the water undigested, however, thereby conferring a survival advantange to the hardy cyanobacterium.

Other factors that favor dominance by *Microcystis* include the cells’ ability to inflate their gas vesicles to rise to the surface of turbid water, where there is plenty of light for photosynthesis. If a cell is running low on phosphorus, its gas vesicles collapse, and it sinks to the bottom where it scavenges this nutrient from the sediments.^5^ Other kinds of phytoplankton lack this ability.

Microcystin toxins act by bonding with protein phosphatase enzymes, especially in liver cells, causing cell damage. The toxins can cause liver and kidney disease in humans who have been exposed through drinking or swimming in contaminated water.^1^ In some cases people have been poisoned via inhalation of microcystins near a major bloom.^1^ In 1996, when a bloom of *Microcystis* poisoned the water supply of a dialysis clinic in Brazil, 56 people died of liver failure.^6^ Blooms producing microcystins have also caused severe and often fatal poisonings of livestock, pets, and wildlife.

## An Initial Focus on Phosphorus

In the 1960s, Lake Erie was choking on excess phosphorus and nitrogen released in poorly treated sewage, industrial waste, and runoff from farm fields and city streets. Diatoms and multiple species of cyanobacteria absorbed the nutrients and flourished; they formed floating mats that shaded underwater plants, inhibiting photosynthesis and causing them to wither away and decompose.^7^ As algal cells died, they sank to the bottom, where they too decomposed. Decomposition takes up oxygen, and the result of the blooms was a dead zone, a span of water so depleted of oxygen that no fish, and few invertebrates, could survive. This phenomenon, called eutrophication, occurs on every continent except Antarctica.^8^

**Figure:**
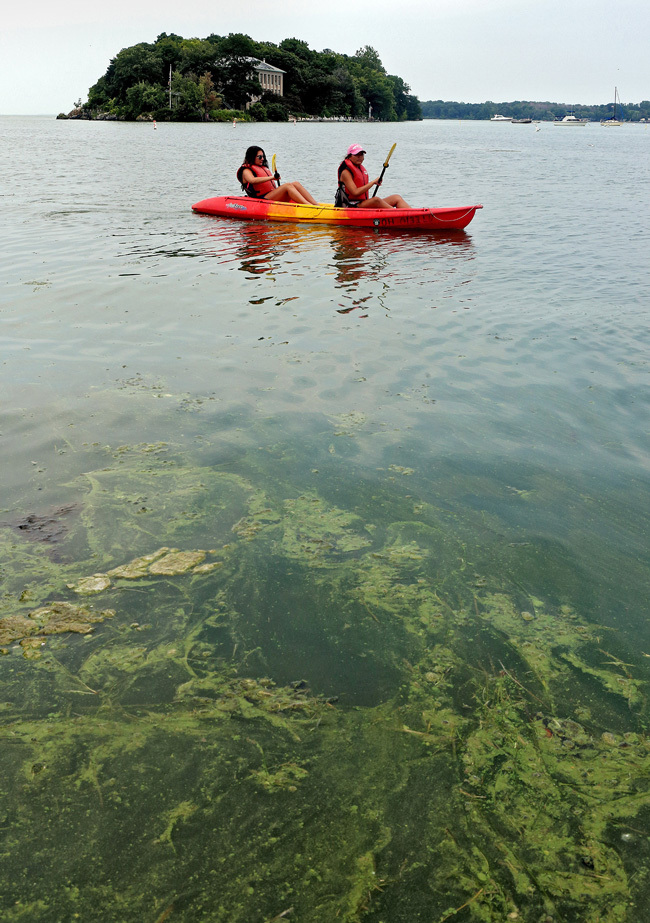
These kayakers paddled on Lake Erie in late July 2015 at the start of what would become the largest *Microcystis* bloom ever documented there. Exposure to *Microcystis* toxins through drinking contaminated water, skin contact, and inhalation can be dangerous, potentially resulting in liver or kidney disease. © Eric Albrecht/The Columbus Dispatch* via AP*

Lake ecologists searched for the key nutrient controlling the growth of HAB-forming species. “Building any living organism is like building a house,” explains Steven Wilhelm, a professor of microbial ecology at the University of Tennessee. “A house needs support beams, doors, and windows. Algae need carbon, nitrogen, and phosphorus. If you don’t have enough of one nutrient, the growth of the algal community is constrained.”

By the late 1960s, researchers suspected that phosphorus was the key nutrient in freshwater blooms.^9^ Later, pioneering experiments led by David Schindler at the Experimental Lakes Area in Canada showed that adding a combination of phosphorus and nitrogen could quickly turn a pristine lake into a green soup, while the addition of carbon showed no effect.^10^ The blooms in the fertilized lakes Schindler studied were dominated by the cyanobacteria *Anabaena* and *Aphanizomenon*. These organisms are capable of converting nitrogen gas to bioavailable ammonia, a process called fixation. Schindler concluded that controlling nitrogen levels in water would not affect the growth of the blooms, since the species involved could take as much nitrogen as they needed out of the air. His work seemed to prove that phosphorus alone was the nutrient of concern.

A ban on phosphate detergents, combined with phosphorus removal programs at wastewater treatment plants, brought a dramatic improvement in Lake Erie’s water quality. In the late 1980s and early 1990s, HABs faded and populations of walleye and other game fish rebounded. But the lake has since slid backward, once again showing signs of chronic nutrient pollution.

An emerging body of research suggests that a failure to control growing loads of both dissolved reactive phosphorus and reactive nitrogen in agricultural and urban runoff has changed the makeup of the HABs that occur there. *Microcystis*—which lacks the ability to fix nitrogen—was present in the twentieth-century lake but was relatively scarce. Today, however, it dominates HABs not only in Lake Erie but in polluted waters around the world, including Florida’s Lake Okeechobee, the Baltic Sea, Lake Taihu in China, and Lake Ohnuma in Japan.

The first extensive bloom of *Microcystis* in western Lake Erie occurred in 1995, and major blooms have since become a predictable summertime event.^11^ Today the nutrient overload primarily comes from agricultural runoff, but urban runoff can also play a role. Both are hard-to-control “non-point” sources of pollution, meaning their release does not come from a specific source, such as a factory. In 2016 the United States and Canada agreed to a new target of a 40% reduction in Lake Erie’s phosphorus loads compared with 2008 levels, aimed at controlling the annual blooms.^12^ But researchers delving into the ecology of *Microcystis* now argue that the traditional approach of focusing on managing loads of total phosphorus ignores the shifting ecology of modern lakes and estuaries.

## Nitrogen: The New Part of the Story

In the 1970s, water quality managers believed that phosphorus ran off agricultural land only in particulate form, attached to bits of sediment. A shift toward no-till farming has succeeded in reducing soil erosion and the release of particulate phosphorus, but has been accompanied by a dramatic rise in dissolved reactive phosphorus,^13^ because fertilizer is spread on the surface of the soil without being tilled under.

**Figure:**
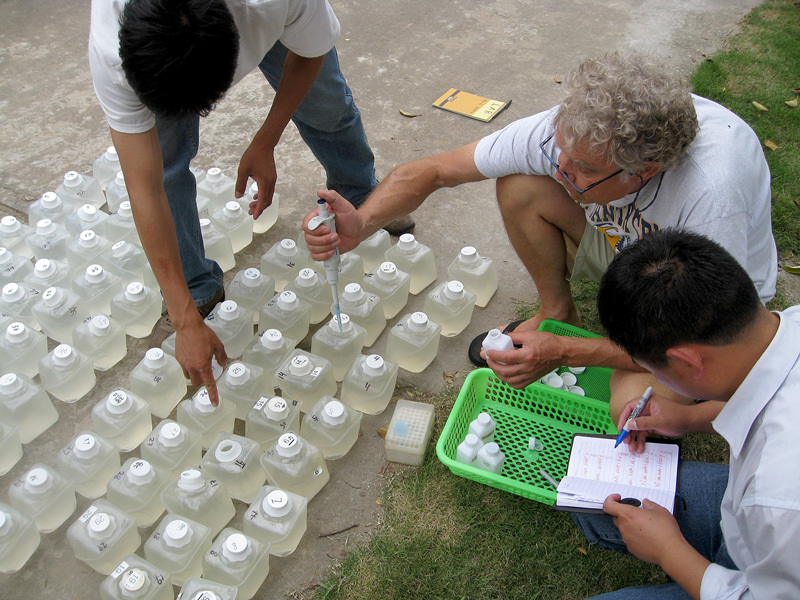
In 2013 Hans Paerl and Chinese colleagues conducted bioassays at Lake Taihu to assess the impact of nutrient enrichment on cyanobacterial blooms. The containers were filled with lake water treated with various concentrations of nitrogen and phosphorus to show how the nutrients affected the lake’s natural algal community. © Hans Paerl/University of North Carolina at Chapel Hill

**Figure:**
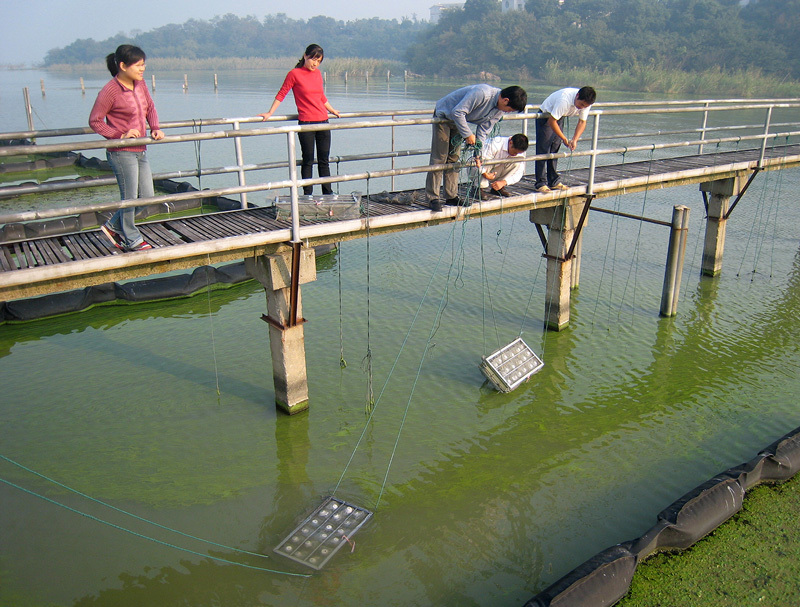
The researchers lowered the bioassay containers into Lake Taihu in frames and left them to incubate under natural light and temperature conditions for up to 4 days. Each day they measured the algal growth response. © Hans Paerl/University of North Carolina at Chapel Hill

Nitrogen also plays a critical role, allowing *Microcystis* to dominate a community of phytoplankton and fueling its production of toxins. The Haber-Bosch process, which uses a catalyst to transform atmospheric nitrogen into bioavailable ammonia, was developed early in the twentieth century. This process enabled mass production of synthetic fertilizers and transformed the global nitrogen cycle.^14^ Large regions of the world now receive nitrogen loads more than an order of magnitude higher than natural rates.^14^ Combustion of fossil fuels also creates significant amounts of bioavailable nitrogen, which settles out of the air into watersheds.^1^

The amount of nitrogen now flowing into many lakes is much greater than it was in the 1970s, when the phosphorus-only paradigm of eutrophication control emerged. In many watersheds, the focus on phosphorus control means that phosphorus levels have stabilized while nitrogen loading continues. The end result is that the balance of nutrients has shifted in many aquatic ecosystems.^15^ In addition, since the 1960s synthetic nitrogen fertilizers in the United States have been based less on ammonium nitrate and more on urea, an organic nitrogen compound that can upregulate microcystin production.^16^

Experiments conducted by Justin Chaffin, Stone Laboratory’s research coordinator, have shown that *Microcystis* blooms begin when the waters are high in nitrogen.^17^ Toxin production occurs early in a bloom; later on, the massive population of cyanobacteria has taken up all the available nitrogen—there’s not enough left to use in producing microcystins, which are nitrogen-rich peptides.^18^ But *Microcystis* is able to maintain high biomass after water levels of nitrogen have been depleted, Chaffin adds, because it is highly competitive for low levels of bioavailable ammonia.

In Lake Erie, the size of each summer’s HAB reflects phosphorus concentrations at the time.^13^ But high levels of nitrogen favor dominance of those blooms by *Microcystis*, and the more nitrogen, the more toxin the bloom will produce.^18^ ^,^ ^19^ In other words, reducing phosphorus levels in Lake Erie will reduce the size of any HAB but not necessarily the amount of toxin produced by *Microcystis* cyanoHABs.

Lake Taihu, in China’s rapidly developing Yangtze River Delta, suffers thick blooms of *Microcystis* from March through November. More than 40 million people live in Taihu’s watershed, and 10 million rely on the lake for their drinking water.^15^

Thirty years ago the lake was relatively clean, its phytoplankton community dominated by benign diatoms. Today Taihu is highly eutrophic. In 2007 the water supply for the lakeside city of Wuxi, then home to more than 2 million people, was disrupted for a full week because of microcystin contamination.^20^

**Figure:**
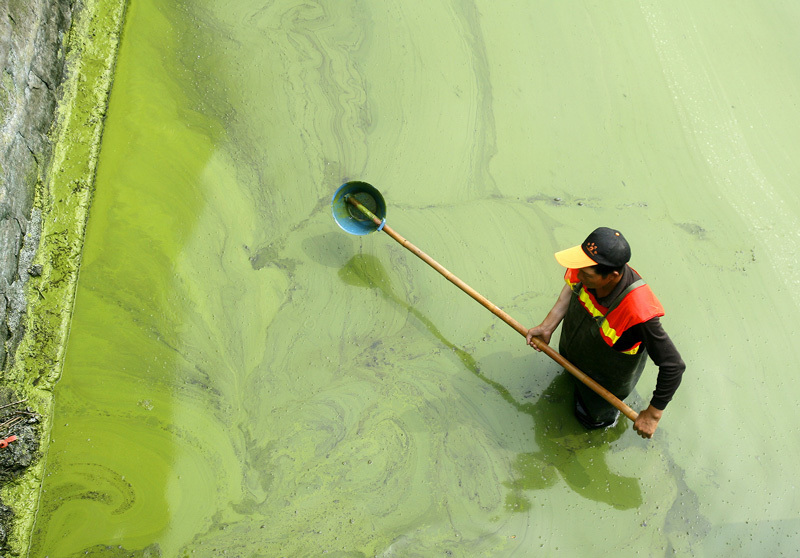
This worker tried to clean up *Microcystis* near a water intake during a major 2007 bloom on Lake Taihu, which shut down the water supply for the city of Wuxi for a week. The lake endures blooms for 9 months out of the year, although not every bloom produces the same amount of toxin. © AFP Photo/Liu Jin

In Taihu, the same problems that affect Lake Erie are magnified because a much larger human population is polluting a shallower, warmer body of water.^15^ *Microcystis* thrives in these conditions.^21^ Lake Taihu’s blooms of *Microcystis* can be intense and at times can cover the entire lake. “It forms a paint-like, iridescent green scum covering the surface,” says Paerl. “It looks like guacamole with a crust.”

That same description has been used in press reports of recent intense *Microcystis* blooms on Lake Okeechobee in Florida, which receives polluted farm and urban runoff, and on the St. Lucie Estuary, where Okeechobee’s waters were diverted after unusually heavy rains beginning in the winter of 2015–2016.^22^ In Okeechobee, as well as Lake Erie, the interaction between nutrient pollution, increased precipitation, and warmer temperature seems to favor the proliferation of *Microcystis*.^1^ ^,^ ^2^

## Shifts in Strategy

In China, as in the United States and other nations, runoff from farm fields is a major driver of eutrophication. “In the watersheds of both Lake Erie and Taihu, steps are being taken to retain fertilizer on the land,” says Paerl. Strategies include the creation of vegetated buffers at the edge of farm ditches and constructed wetlands that filter nutrients from runoff.

These techniques can capture both nitrogen and phosphorus, but to solve the problem of non-point nutrient pollution, they may have to be deployed on a large scale that would cover a significant fraction of the landscape. In 2005 William Mitsch, who is eminent scholar and director of the Everglades Wetland Research Park, estimated that 22,000 square kilometers of land would need to be restored to wetland in the Mississippi River Basin to remove 40% of the nitrogen flowing to the Gulf of Mexico.^23^ In Midwest farmlands, he says today, 7–10% of the landscape would need to be restored to wetlands to capture nutrients flowing off the remaining 90–93%.

Davis notes that regulators are beginning to come to grips with the role of nitrogen in eutrophic lakes. Researchers who guide updates to the Great Lakes Water Quality Agreement,^24^ a pact between the United States and Canada, are preparing a report on the need to increase the understanding of nitrogen pollution and cycling in Erie and the other Great Lakes.

In 2015 the U.S. Environmental Protection Agency published a report on the need to consider both nitrogen and phosphorus in water quality regulations.^25^ That report noted that a focus on phosphorus control has not resolved problems in many freshwater bodies, that marine habitats are impacted by heavy loads of nitrogen flowing downstream, and that the relationship between bioavailable nitrogen and cyanoHABs is far more complex than was recognized in the 1970s. “The weight of scientific evidence,” the authors concluded, “supports the development of nutrient criteria for both [nitrogen and phosphorus].”^25^

If the rise of *Microcystis* highlights the long-ignored role of nitrogen in eutrophication of fresh waters, it also underscores the difficulty of controlling non-point source water pollution, a task currently managed by each state. Multiple scientists and farmers interviewed for this story indicate that the task of managing non-point source pollution is complicated, because many landowners resent government prescriptions as to how they manage their farms—particularly if they are required to take cropland out of production to create vegetated buffer zones or restore wetlands.

**Figure:**
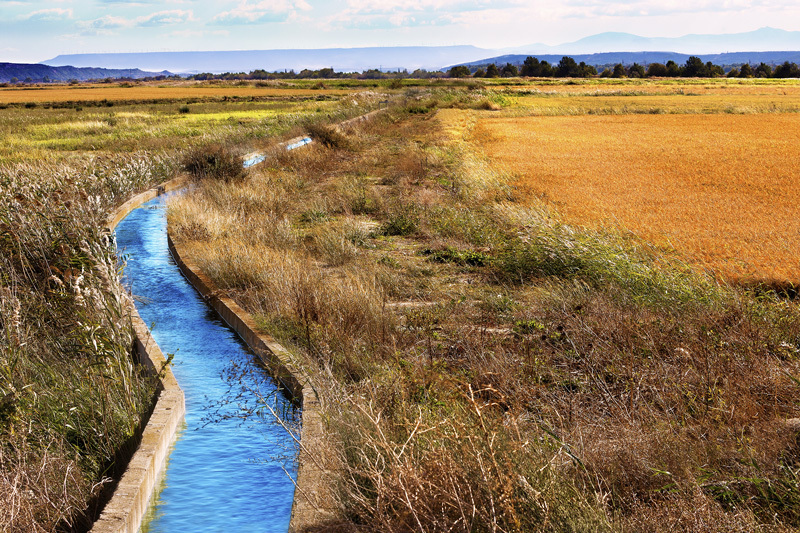
Buffers of undisturbed vegetation along streams and canals are one way to absorb excess pollutants from runoff before they reach larger water bodies, where they contribute to blooms. © Carlos Castilla/Shutterstock

Federal programs offer subsidies to farmers who voluntarily create buffer strips or constructed wetlands on their property.^26^ A recent modeling study by University of Michigan researchers suggests, however, that reaching the goal of a 40% reduction in Lake Erie’s phosphorus load will take more widespread use and strategic placement of nutrient capture strategies.^27^ At this point, little is known about the patterns of nitrogen cycling in the Lake Erie watershed.

Major cleanup efforts of point sources of pollution have, in many cases, achieved only a short intermission in the ongoing process of eutrophication. Humanity continues to alter nutrient cycles in ways that affect aquatic habitats worldwide. Rescuing lakes and estuaries from non-point source nutrient pollution will be a long, complex struggle—and success will depend on our awareness of such mundane acts as dropping fertilizer onto soil, as well as our understanding of tiny but powerful organisms like *Microcystis*.
