# Erratum: “*Microcystis* Rising: Why Phosphorus Reduction Isn’t Enough to Stop CyanoHABs”

**DOI:** 10.1289/EHP1787

**Published:** 2017-03-31

**Authors:** Sharon Levy

Environ Health Perspect 125(2):A34–A39 (2017), http://dx.doi.org/10.1289/ehp.125-A34

One of the researchers quoted in this article was not fully identified. Timothy Davis is a research scientist at the Great Lakes Environmental Research Laboratory of the National Oceanic and Atmospheric Administration.

*EHP* regrets the error.

